# Brief early life angiotensin-converting enzyme inhibition attenuates the diuretic response to saline loading in sheep with solitary functioning kidney

**DOI:** 10.1042/CS20230663

**Published:** 2023-08-22

**Authors:** Zoe McArdle, Reetu R. Singh, Karen M. Moritz, Michiel F. Schreuder, Kate M. Denton

**Affiliations:** 1 Cardiovascular Program, Monash Biomedicine Discovery Institute and Department of Physiology, Monash University, Melbourne, Australia; 2 Child Health Research Centre and School of Biomedical Sciences, The University of Queensland, Brisbane, Queensland, Australia; 3 Department of Pediatric Nephrology, Amalia Children’s Hospital, Radboud University Medical Center, Nijmegen, The Netherlands

**Keywords:** angiotensin converting enzyme inhibition, sheep, sodium homeostasis, Solitary functioning kidney, water homeostasis

## Abstract

A solitary functioning kidney (SFK) from birth predisposes to hypertension and kidney dysfunction, and this may be associated with impaired fluid and sodium homeostasis. Brief and early angiotensin-converting enzyme inhibition (ACEi) in a sheep model of SFK delays onset of kidney dysfunction. We hypothesized that modulation of the renin–angiotensin system via brief postnatal ACEi in SFK would reprogram renal sodium and water handling. Here, blood pressure (BP), kidney haemodynamics and kidney excretory function were examined in response to an isotonic saline load (0.13 ml/kg/min, 180 min) at 20 months of age in SFK (fetal unilateral nephrectomy at 100 days gestation; term 150 days), sham and SFK+ACEi sheep (ACEi in SFK 4–8 weeks of age). Basal BP was higher in SFK than sham (∼13 mmHg), and similar between SFK and SFK+ACEi groups. Saline loading caused a small increase in BP (∼3–4 mmHg) the first 2 h in SFK and sham sheep but not SFK+ACEi sheep. Glomerular filtration rate did not change in response to saline loading. Total sodium excretion was similar between groups. Total urine excretion was similar between SFK and sham animals but was ∼40% less in SFK+ACEi animals compared with SFK animals. In conclusion, the present study indicates that water homeostasis in response to a physiological challenge is attenuated at 20 months of age by brief early life ACEi in SFK. Further studies are required to determine if ACEi in early life in children with SFK could compromise fluid homeostasis later in life.

## Introduction

Mounting evidence indicates that a congenital solitary functioning kidney (SFK) is associated with hypertension and kidney dysfunction from early in life [1–4]. Recently, it has been shown that by 18 years of age ∼75% of children with a congenital SFK show signs of kidney injury and ∼39% severe kidney injury [1]. Additionally, ∼30% of children born with a SFK reach kidney failure by 30 years of age [3]. Tubular sodium and water retention are suggested to contribute to hypertension and kidney dysfunction in models of renal mass reduction [5,6]. Therefore, disturbances in fluid and electrolyte homeostasis may in part underlie the onset of hypertension and kidney disease in SFK.

We have a sheep model of congenital SFK, generated by fetal unilateral nephrectomy at 100 days gestation (term: 150 days) [7]. The removal of one kidney at 100 days gestation in this model likely corresponds to the start of active nephrogenesis in sheep, which is week 26 gestation in humans [8]. In a previous study, we showed blood pressure and kidney function were similarly affected by a high-salt diet in female sheep with a SFK and sham animals [9]. However, in response to volume expansion by acute saline loading, SFK sheep have altered sodium handling compared with sham controls [10–12]. At 6 months of age sheep with a SFK exhibit an increased natriuretic and diuretic response to saline loading compared with sham counterparts [11]. Comparatively, in response to saline loading, 5-year-old sheep with a SFK exhibit a blunted diuretic and natriuretic response to saline loading compared with sham counterparts [10]. This indicates that with age the ability to respond to physiological challenges, specifically changes in fluid and sodium balance, is reduced in SFK sheep [10–12]. Similarly, an exaggerated naturesis and diuresis in response to saline loading is observed in humans with essential hypertension compared with normotensive counterparts [13], with sodium handling declining with age [14].

The renin–angiotensin system (RAS) is an important regulator of fluid and electrolyte homeostasis. In response to a saline load, suppression of renin secretion is a significant stimulus in mounting a robust natriuretic response [15–17]. Renin secretion is modulated by alterations in renal sympathetic nerve activity, renal perfusion pressure and sodium delivery to the macula densa [18]. Therefore, the response to saline loading requires integrated actions of multiple systems to mount a diuretic and natriuretic response. In previous studies in this ovine model of SFK the impaired renal excretory responses to isotonic saline loading in SFK sheep was associated with reduced suppression of PRA [10–12].

Angiotensin-converting enzyme inhibition (ACEi) is an effective first-line treatment for hypertension and kidney disease [19]. Evidence in rodent models of hypertension have shown that brief postnatal ACEi can reduce the onset of hypertension and offer reno-protection long term [20–22]. We have recently shown that a short period of ACEi early in the postnatal period (4–8 weeks of age) in SFK sheep reduces albuminuria independent of blood pressure lowering, improves kidney hemodynamics, renal functional reserve and nitric oxide bioavailability at 8 months of age [23]. Although these reno-protective benefits waned with age, with albuminuria similar to untreated SFK levels at 20 months, there were residual kidney functional changes still present 18 months after the withdrawal of ACEi [24].

The purpose of the present study was to examine whether modulation of the RAS via ACEi (enalapril, 0.5 mg/kg/day, orally) for 4 weeks between 4 and 8 weeks of age in SFK sheep altered the diuretic and natriuretic responses to slow volume expansion at 20 months of age, 18 months after treatment withdrawal.

## Methods

### Animals

All experimental procedures were approved by an Animal Ethics Committee of Monash University (Ethics numbers: MARP/182/2016, #20442) and were performed in accordance with the guidelines laid down by the National Health and Medical Research Council of Australia. All sheep were housed at the Monash University’s Gippsland Field Station in between surgical/experimental procedures and at Monash Animal Research Platform during surgical/experimental procedures. A detailed description of the surgical procedures has previously been published [23], but it should be noted that all surgical procedures were performed under isoflurane anaesthesia. In brief, a congenital SFK was generated by performing unilateral nephrectomy in the sheep fetus at 100 days gestation (term = 150, SFK, *n*=19) or sham surgery was performed (sham, *n*=9), as previously described [23,25]. Only males were used in this study. Following recovery (∼10 days), the ewes were returned to the farm to lamb down, with birth weights previously reported [24]. Lambs from the SFK group (*n*=19) were randomly assigned to undergo ACEi treatment at 4 weeks of age, via administration of enalapril (maleate salt, E6888) once daily orally (*n*=10, 0.5 mg/kg/day, SFK+ACEi group), which was stopped at 8 weeks of age. The remaining animals received vehicle (water, SFK, *n*=9). At 6 months of age, lambs had surgery to isolate and exteriorise their carotid arteries into a skin fold to form carotid arterial loops, which allowed access to the carotid artery for catheterization and blood sampling [7].

### Experimental protocol

At 20 months of age sheep were individually housed in metabolic cages and offered water and 800 g of chaff once daily. Intake of food and output of water and urine were monitored daily. The animals underwent surgery for insertion of a bladder catheter, as previously detailed [11], following 3 days of housing in the metabolic cage. After appropriate recovery (3–4 days) and prior to the experimental period, under local anaesthetic (subcutaneous, 20 mg/kg, lignocaine with 0.5% adrenaline) sheep were instrumented with an indwelling carotid arterial catheter (PVC tubing, 1.5 × 1.0 mm, Microtube Extrusions, Australia) that was connected to a pressure transducer (Argon pressure transducer, LivaNova Australia Pvt. Ltd.) to allow continuous measurements of blood pressure (BP) (systolic, diastolic, mean arterial pressure [MAP]) and heart rate (HR), as previously described [26]. In addition, a polyethylene catheter was introduced into the jugular vein (PE tubing, 1.2 × 1.0, Microtube Extrusions, Australia) for infusion purposes. At 20 months of age each sheep underwent three separate studies: (1) vehicle infusion (time control study), previously reported as a singular average [24], (2) renal functional reserve study previously reported [24], and (3) isotonic saline load. Due to COVID-19 restrictions at the time of experimentation, we were unable to perform the saline experiment in *n*=3 animals (sham, *n*=1; SFK+ACEi, *n*=2); for continuity, all results are presented excluding these animals.

### Vehicle infusion (time control study)

The day following catheterisation, sheep underwent a 7-h time-control study to examine basal cardiovascular and kidney function over time. ^51^Chromium ethylenediaminetetraacetic acid (^51^Cr EDTA) and p-aminohippuric acid (PAH) were administered (i.v.) at a combined rate of 12 ml/h for the determination of glomerular filtration rate (GFR) and renal blood flow (RBF), respectively, via clearance methods as previously described [10]. Following 1 h of equilibration, urine was collected every 20 min and blood samples (3 ml) collected at every mid-point of each urine collection. Simultaneously, both BP (systolic, diastolic and MAP) and HR were measured continuously every 10 s, with data reported as hourly averages.

### Isotonic saline loading

On a separate day, the cardiovascular, kidney hemodynamic and excretory responses to isotonic saline infusion (0.9% sodium chloride, 154 mmol/l, Baxter Healthcare, U.S.A.) for 180 min was examined. Following one hour of equilibration, urine samples were collected every 20 min throughout the study with an arterial blood sample (3 ml) taken at mid-point of each urine collection. Baseline data were collected over a period of 60 min. Then isotonic saline infusion was commenced at 0.13 ml/kg/min, which equates to 20 µmol Na^+^/kg/min [27], for 180 min (i.v) followed by a 180-min period of recovery. During this period both BP (systolic, diastolic and MAP) and HR were measured continuously. In addition, arterial blood samples (5 ml) were collected on EDTA at the end of the baseline, saline load and recovery periods for the assessment of PRA. Within 2–3 days of experimentation, sheep were killed with an overdose of pentobarbitone sodium (100 mg/kg, iv.).

### Sample analysis

^51^Cr EDTA levels were measured using a gamma counter (PerkinElmer Wizard 1470). PAH concentration was determined using a using a previously described rapid microplate assay method [28] and effective renal plasma flow (ERPF) and RBF (ERPF)/(1-hematocrit) calculated. Urinary sodium concentration was measured (Beckman Coulter, Monash Medical Centre) and corrected for urine flow (urinary sodium excretion; U_Na_V). PRA was determined by radioimmunoassay (Prosearch International Pty, Malvern, Australia). Renal vascular resistance (RVR) was calculated as (MAP/RBF), and filtration fraction was calculated as (GFR/ERPF).

### Immunohistochemistry for renal sympathetic nerves tyrosine hydroxylase

After euthanasia, kidneys were flushed with saline, immersion fixed in 4% paraformaldehyde and embedded in paraffin and sectioned (4 µm) (Histology Platform, Monash University). Antigen retrieval was performed by treating sections with citrate buffer (DAKO) for 30 min at 98°C (Monash Histology Platform). Endogenous peroxidase activity was quenched (0.3% hydrogen peroxide) and non-specific binding blocked with 10% goat serum (1:10 dilution, Vector Laboratories). Slides were incubated in a rabbit anti-tyrosine hydroxylase primary antibody (1:800, AB152, Merck Millipore) at 4°C overnight. The next day, sections were incubated in a biotinylated goat anti-rabbit IgG (H+L) secondary antibody (1:200 dilution, Vector Laboratories) and then in the Avidin-Biotin complex (ABC kit, Vector laboratories). Color was developed with 3,3′-diaminobenzidine (DAB kit, Vector laboratories) and then counterstained with haematoxylin, cover slipped and imaged. Quantification of tyrosine hydroxylase was performed as previously detailed [23].

### Statistical analysis

All values are presented as mean ± S.E.M. Statistical analysis was performed using Graphpad Prism 9.0 (Graphpad software Inc., CA, U.S.A.) and statistical significance was accepted as *P*≤0.05. Data were tested for normality using a Shapiro–Wilk test and were determined to fit Gaussian distribution. An analysis of variance was performed examining the effects of two factors; group (*P*_group_; sham, SFK or SFK+ACEi) and time (*P*_time_) and their interactions for the time control experiment. Similarly, an analysis of variance was performed examining the effects of two factors; group (*P*_group_; sham, SFK or SFK+ACEi) and saline (*P*_saline_ basal and after saline) and their interactions for the saline loading experiment. For the amount of saline infused and excreted and immunohistochemistry a one-way analysis of variance was used and where appropriate a Dunnett’s post-hoc analysis was performed (compared with SFK group).

## Results

### Time control experiment

MAP, HR, GFR, RVR and filtration fraction did not differ significantly over the 7-h period of measurement (Supplementary Figure S1). However, an overall effect of time on RBF was detected (*P*_time_ = 0.02, Supplementary Figure S1d), post-hoc analysis revealed no significant difference in RBF from baseline in all groups.

### Responses to saline loading

#### Cardiovascular responses to saline loading

**Figure 1:**
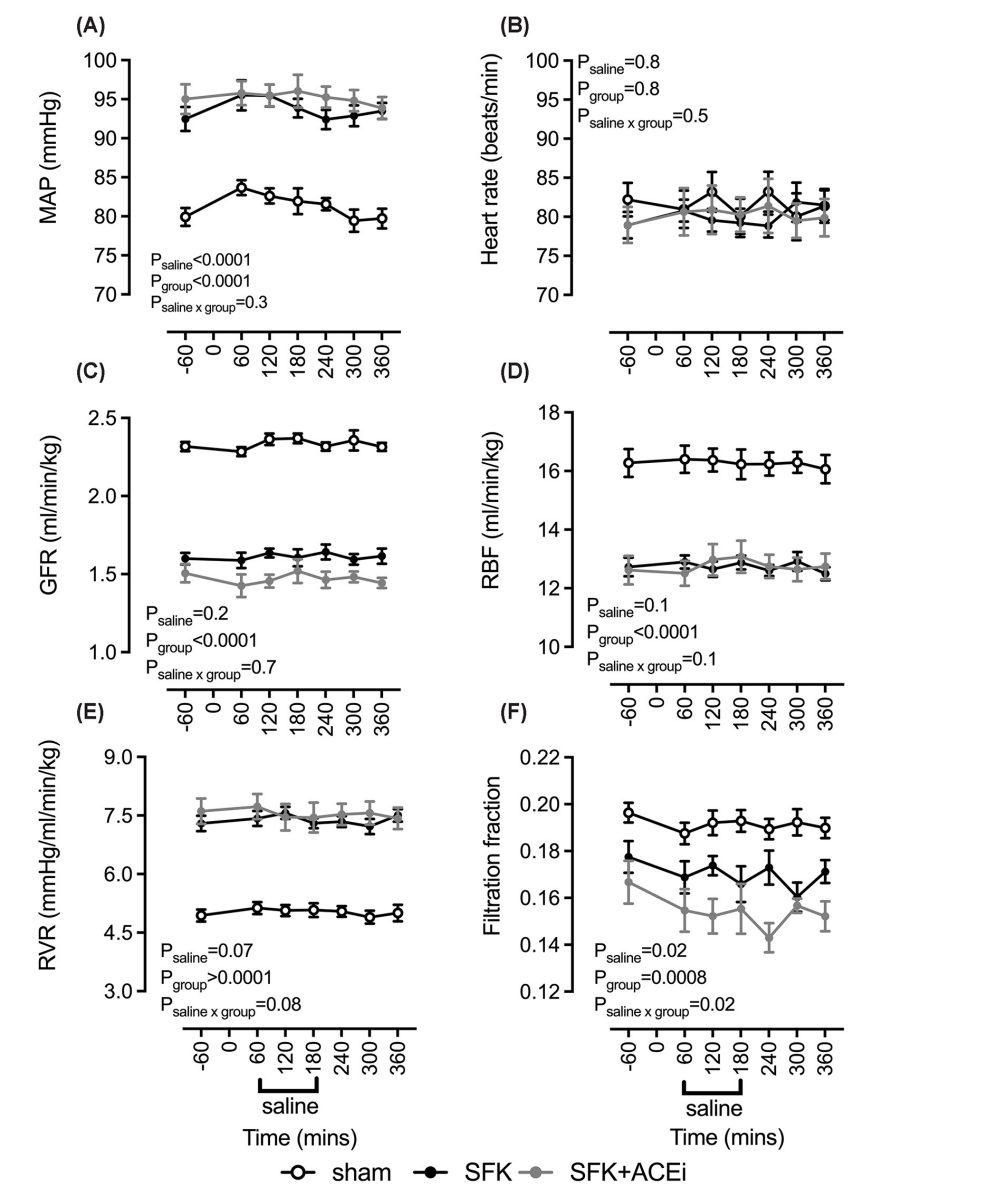
Cardiovascular (A,B) and kidney hemodynamics (C–F) in response to isotonic saline loading Data presented in male lambs that underwent fetal sham surgery (*n*=8), fetal uninephrectomy (SFK, *n*=9) or fetal uninephrectomy and (ACEi via enalapril between 4 and 8 weeks of age (SFK+ACEi;* n*=8). Data analyzed via a two-way repeated measures analysis of variance. GFR, glomerular filtration rate; MAP, mean arterial pressure; RBF, renal blood flow; RVR, renal vascular resistance.

On the day of saline loading, basal MAP was higher in the SFK group compared with the sham group (∼13 mmHg, *P*<0.0001, Figure 1A) but was similar between SFK and SFK+ACEi groups. In response to saline loading, MAP increased from baseline (*P*_saline_<0.0001, Figure 1A). In SFK and sham groups, MAP was significantly higher than baseline for the first 2 hours of saline loading and then returned to baseline levels (60–120 min: sham: ∼4 mmHg, *P*<0.05; SFK: ∼3 mmHg, *P*<0.05, Figure 1A). In the SFK+ACEi group, saline loading had no significant effect on MAP (Figure 1A). Basal HR was similar between the groups and saline loading had no significant effect on HR (Figure 1B).

#### Kidney hemodynamic responses to the saline loading

Baseline GFR (∼23%) and RBF (∼22%) were significantly lower in the SFK group compared with the sham group (both *P*<0.0001, Figure 1C,D). Compared with sham sheep, RVR was ∼47% higher in SFK sheep (Figure 1E). Baseline filtration fraction was similar between SFK and sham sheep (Figure 1F). Basal GFR, RBF, RVR and filtration fraction were not different between SFK and SFK+ACEi groups (Figure 1C–F).

The saline load did not alter GFR (*P*_saline_=0.2), RBF (*P*_saline_=0.1) or RVR (*P*_saline_=0.07) in any group (Figure 1C–E). An overall effect of saline loading on filtration fraction was observed (*P*_saline_=0.02, Figure 1F) and the response to saline loading differed in the groups (*P*_saline ×group_=0.02, Figure 1F). Saline loading caused filtration fraction to be lower than baseline at 300 min in SFK animals (*P*=0.049) and at 120 (*P*=0.04), 240 (*P*=0.0001) and 360 min (*P*=0.04) in SFK+ACEi animals.

#### Plasma renin activity response to saline loading

**Figure 2:**
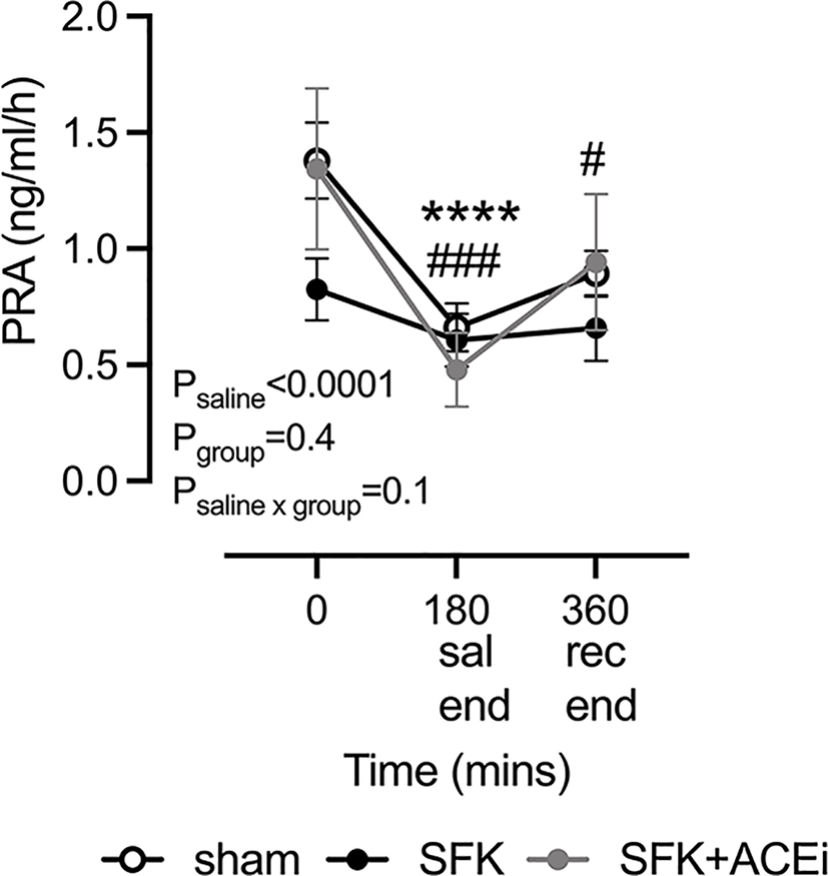
PRA in response to isotonic saline loading Data presented in male lambs that underwent fetal sham surgery (*n*=8), fetal uninephrectomy (SFK, *n*=9) or fetal uninephrectomy and ACEi via enalapril between 4 and 8 weeks of age (SFK+ACEi, *n*=8). Data analyzed via two-way repeated measures analysis of variance. Data are mean ± S.E.M. (**A**) #*P*<0.05, ###*P*<0.001, compared with baseline for sham, ****P<0.0001 compared with baseline for SFK+ACEi from post-hoc Dunnett’s test. Sal end; saline end (180 min), rec end; recovery end (360 min).

Basal PRA was ∼40% lower in SFK sheep compared with sham sheep (*P*=0.04, Figure 2) but was not significantly different between SFK and SFK+ACEi sheep. Overall, there was an effect of saline loading on PRA (*P*_saline_<0.0001, Figure 2). Saline loading caused PRA to decline from baseline in sham (*P*=0.0008, Figure 2) and SFK+ACEi groups (*P*<0.0001, Figure 2) but not in the SFK group (Figure 2). At the end of the recovery period, PRA was not restored to baseline levels in sham animals (*P*=0.02) but was in SFK+ACEi animals (*P*=0.068, Figure 2).

#### Urine flow and sodium excretion responses to saline loading

**Figure 3:**
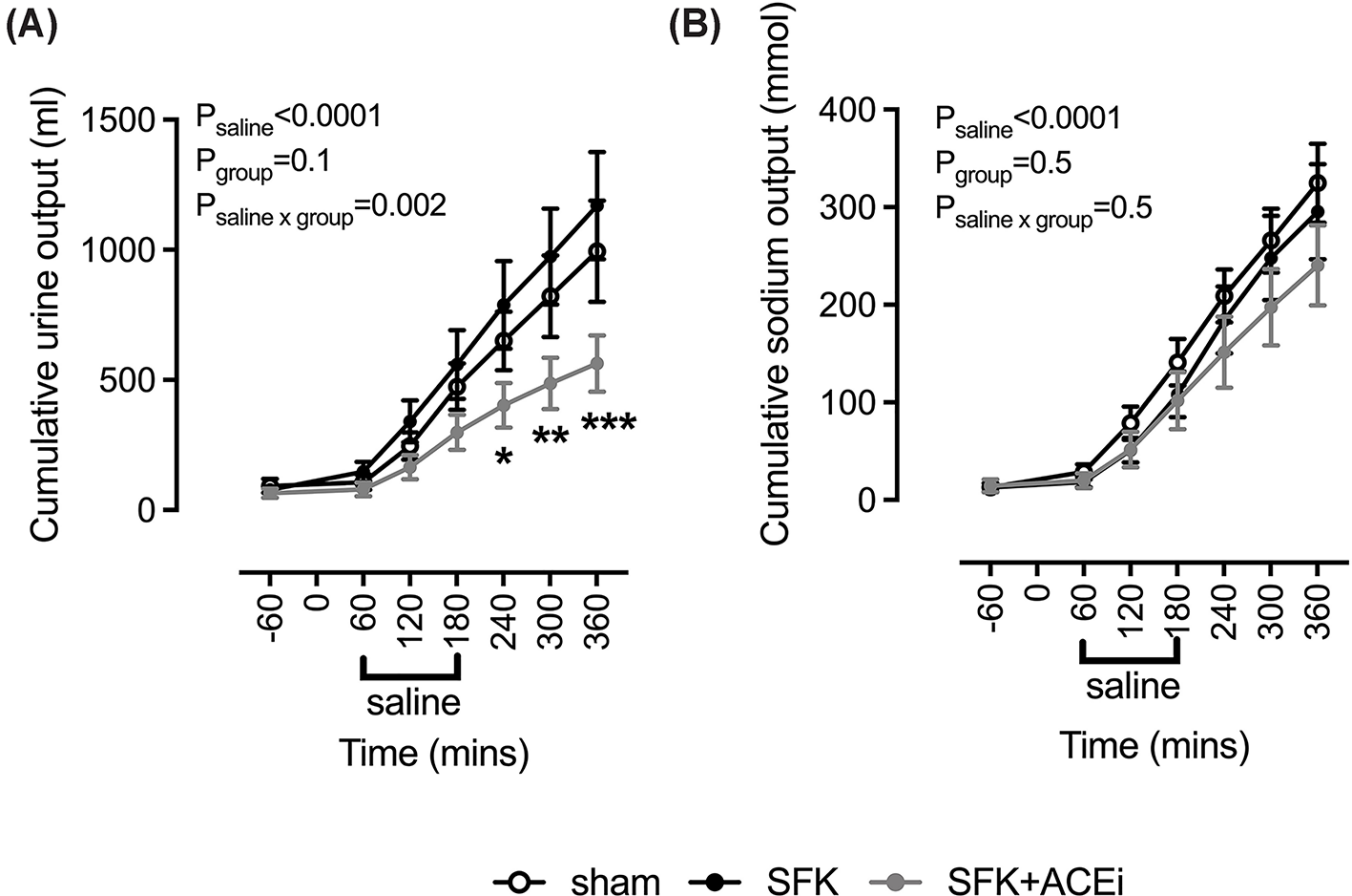
Cumulative urine (A) and sodium output (B) in response to isotonic saline loading Data presented in male lambs that underwent fetal sham surgery (*n*=8), fetal uninephrectomy (SFK, *n*=9) or fetal uninephrectomy and ACEi via enalapril between 4 and 8 weeks of age (SFK+ACEi, *n*=8). Data analyzed via a two-way repeated measures analysis of variance. Data are mean ± S.E.M. (**A**) *P<0.05, **P<0.01, ***P<0.001, comparing with SFK with SFK+ACEi from post-hoc Dunnett’s test.

Basal urine flow and U_Na_V were similar between groups. In response to the saline load cumulative urine output was similar in the SFK and sham animals (Figure 3A). There was a significantly lower increase in cumulative urine output in the recovery period following the saline load in SFK+ACEi animals compared with SFK counterparts (Figure 3A). In response to the saline load cumulative urinary sodium excretion was similar between groups (Figure 3B).

The volume of saline (0.9% NaCl) and sodium (Na^+^) infused was equivalent in all groups (Table 1). Neither the sham, SFK or SFK +ACEi sheep excreted the full saline load by the end of the 3-h recovery period (Table 1). Both SFK and sham sheep excreted a similar percentage of the total volume saline infused (Table 1). Comparatively, the SFK+ACEi sheep excreted significantly less percentage of the total volume infused compared with SFK counterparts (Table 1). Expressed as a percent of sodium infused, sodium output was similar between sham and SFK and similar between SFK and SFK +ACEi sheep (sham, 150 ± 19%; SFK, 127 ± 19%; SFK+ACEi, 104 ± 18%; Table 1).

**Table 1:** Total volume and sodium infused and excreted in response to isotonic saline loading over 180-min infusion plus 180-min recovery period

	Sham (*n*=8)	SFK (*n*=9)	SFK+ACEi (*n*=8)
Body weight (kg)	61 ± 3	64 ± 2	65 ± 2
Total volume infused (ml)	1427 ± 72	1488 ± 51	1529 ± 51
Total sodium infused (mmol)	220 ± 11	229 ± 8	236 ± 8
Total volume excreted (ml)	994 ± 194	1170 ± 206	563 ± 108*
Total sodium excreted (mmol)	325 ± 41	296 ± 49	241 ± 41
Total volume excreted (as percent of saline load infused)	68 ± 11	79 ± 14	38 ± 8*
Total sodium excreted (as percent of saline load infused)	150 ± 19	127 ± 19	104 ± 18

*P<0.05, comparing SFK and SFK+ACEi groups from post-hoc Dunnett's test following one-way ANOVA.

### Immunohistochemical staining for tyrosine hydroxylase

**Figure 4:**
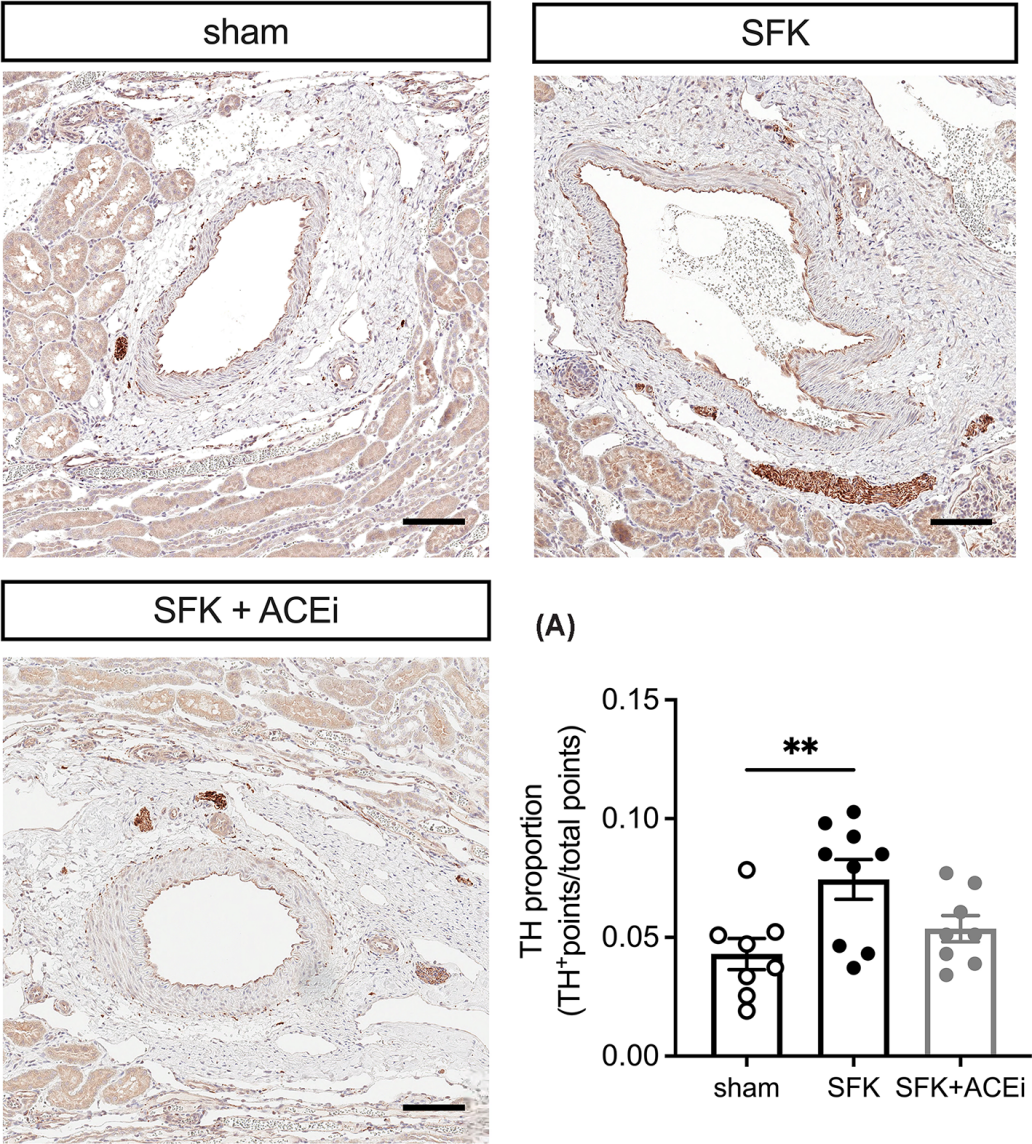
Representative micrograph images of immunohistochemistry staining of tyrosine hydroxylase (TH) and TH proportion in arcuate arteries at 20 months of age (A) TH (dark brown) staining efferent renal sympathetic nerves of the arcuate arteries (scale bar in 100 μm [black]). Data presented in male lambs that underwent fetal sham surgery (*n*=8), fetal uninephrectomy (SFK, *n*=9) or fetal uninephrectomy and ACEi via enalapril between 4 and 8 weeks of age (SFK+ACEi, *n*=8). *P-*values are from Dunnett’s post-hoc test following one-way analysis of variance. **P<0.01, comparing between sham vs. SFK groups.

The proportion of TH was greater in SFK sheep compared with sham animals (*P*=0.009, Figure 4) and similar between SFK and SFK+ACEi sheep (*P*=0.086, Figure 4).

## Discussion

The present study showed that the excretion of sodium and fluid volume in response to a slow isotonic saline load was similar between sham and SFK groups at 20 months of age. In comparison, brief postnatal ACEi in SFK sheep resulted in a blunted diuretic but not natriuretic response to isotonic saline loading compared with SFK sheep at 20 months of age. Brief postnatal ACEi in SFK sheep also resulted in a restored PRA response to saline loading compared with that of SFK. Taken together, these data indicate that the kidney concentrating system in response to a physiological challenge is attenuated by early life ACEi in SFK sheep, 18 months following the withdrawal. This may indicate brief early life ACEi has adverse consequences for fluid homeostasis; alternatively, it could reflect an improved ability to conserve water in the long-term.

In response to the stimulus of extracellular fluid volume expansion, the contribution of different elements of the natriuretic and diuretic responses may be revealed [29]. A rapid saline infusion triggers an increase in blood pressure as well as RBF and GFR [10,11,29]. Comparatively, a slow isotonic saline load triggers a lesser increase in blood pressure and does not alter RBF and GFR [15–17]. Due to the differences in cardiovascular and kidney haemodynamic responses to the rate of saline load, the underlying mechanisms driving the increase in sodium and volume excretion are different. In order to reduce the confounding effects of cardiovascular and kidney hemodynamic changes with a rapid saline infusion, we utilized a slow isotonic saline infusion. In the present study, the excretion of sodium and volume in response to slow isotonic saline loading was comparable in SFK and sham sheep. In a previous study, in response to slow isotonic saline loading, SFK sheep aged 12 months had a greater sodium excretion than sham animals but by 21 months of age (similar to the present study), sodium excretion in response to volume loading was similar between the SFK and sham animals [12]. Additionally, in response to rapid infusion of isotonic saline, SFK sheep aged 6 months had exaggerated natriuretic and diuretic responses [11] whereas the natriuretic and diuretic responses in 5-year-old SFK sheep were blunted [10]. Collectively, the natriuretic and diuretic responses to both rapid and slow volume expansion in this model of SFK indicate that these responses are impacted by age [10]. In contrast, in SFK sheep treated with an ACEi early in life the ability to excrete the volume, but not the sodium, was blunted. This suggests that early life ACEi may have reprogrammed the vasopressin system, an action that has persisted to 20 months of age. Future studies are required to determine whether early life interventions in the RAS have a long-lasting impact on fluid homeostasis and if kidney excretory function declines further with ageing in SFK sheep following ACEi.

Brief early life ACEi in SFK sheep also caused a greater magnitude of PRA suppression in response to saline loading compared with SFK counterparts. The attenuation of PRA is an important stimulus for the increase in sodium excretion in response to saline loading [16,17]. Therefore, this may indicate that although SFK+ACEi sheep manage to excrete sodium to the same degree as the SFK sheep in response to slow isotonic saline loading, the mechanism by which this is achieved is likely different. A difference in basal PRA level was not detected between SFK and SFK+ACEi groups, but in the SFK+ACEi sheep PRA declined significantly, whereas PRA did not in the SFK group. The mechanisms underlying the greater suppression of PRA in response to saline loading in SFK+ACEi sheep are unclear. Moreover, the restoration of PRA levels despite the slower excretion of the saline load suggests that regulation of renin release may have been affected by early life ACEi. Renin secretion is suppressed by multiple mechanisms including a reduction in renal sympathetic stimulation of juxtaglomerular β-1 adrenoreceptors, increase in renal perfusion pressure and increase in NaCl delivery to the macula densa (MD) [18]. However, blockade of β-1 adrenoreceptors during slow isotonic saline loading in dogs did not yield inhibition of the plasma renin response [16,17], suggesting that this mechanism may also not be of importance in the current study. This is further supported by the similar level of tyrosine hydroxylase proportion in SFK and SFK+ACEi animals. In addition, in the present study, MAP was unchanged by saline infusion in SFK+ACEi animals and therefore changes in renal perfusion pressure likely did not contribute substantially to the greater level of PRA suppression in these animals [30]. Isotonic saline infusion has been demonstrated to result in diminished sodium reabsorption at the proximal tubule [31], which can lead to enhanced delivery of NaCl to the distal tubule independent of alterations in whole kidney GFR [32], thus reducing renin secretion facilitating sodium excretion.

In the present study, the SFK sheep exposed to ACEi early in life excreted a similar percent of total sodium but ∼39% less total volume of the slow isotonic saline load as compared to the SFK. The mechanisms underlying the dissociation of the diuretic and natriuretic responses to saline loading in SFK+ACEi sheep are unclear. However, there is evidence in humans [33] and mice [34] that an increase in dietary salt can lead to enhancement of sodium excretion without a major increase in urine volume, which is the result of a complex enhancement of urea osmolyte production and recycling to conserve body water. Whether an improved ability to conserve water is advantageous in SFK+ACEi sheep is unclear. Alternatively, given arginine vasopressin (AVP) is critical for the modulation of water excretion [35], there may be a disruption in the AVP – vasopressin V2 receptor (V2R) – aquaporin 2 (AQP2) axis in SFK+ACEi sheep 18 months after the withdrawal of ACEi. Volume expansion induced stimulation of both right atrial cardiopulmonary [36,37] and arterial baroreceptors [38] are associated with inhibition of AVP release [17,39] facilitating a rise in urine flow. In the present study, there was a small but significant increase in MAP over the first 2 h of saline loading in SFK and sham sheep, which likely increased the loading of arterial baroreceptors. In comparison, MAP was unchanged during the saline load in SFK+ACEi animals, which may have resulted in incomplete suppression of AVP via lesser baroreceptor stimulation thus mediating the greater water retention in response to saline loading in these animals. Both plasma AVP and copeptin levels are challenging to measure in sheep [40], especially in response to stimuli that depress these factors. Given the 2–3 mmHg increase in blood pressure in response to the saline load that occurred in the sham and SFK groups but not in SFK+ACEi group, early life ACEi in SFK may have caused a subtle shift in the pressure natriuresis relationship contributing to the delayed excretion of the saline load. In addition, in the collecting duct angiotensin II (Ang II) can enhance AQP2 trafficking and expression via angiotensin type 1 receptor (AT1R) and via V2R, thus enhancing water permeability in the collecting duct [41,42]. In sheep with SFK both V2R and AQP2 kidney gene expression are lower compared with sham counterparts [43], which is likely associated with the lower level of kidney AT1R expression in SFK sheep [44]. In mice with lithium induced nephrogenic diabetes or unilateral ureteral obstruction, models that are associated with reduced AQP2 kidney expression, renin inhibition for 7 days has been demonstrated to increase AQP2 expression [45,46]. Therefore, if SFK+ACEi animals had enhanced AQP2 apical membrane abundance, this may be one of the mechanisms responsible for the blunting of the diuretic response to saline loading in SFK+ACEi animals.

## Limitations

A limitation of the present study is that a sheep model of SFK, generated by the surgical removal of one kidney at 100 days gestation, cannot recapitulate the variation in the cause or timing of a kidney loss that occurs in humans that are born with a single kidney. In addition, the saline loading study was only performed at 20 months of age, which was 18 months after the withdrawal of ACEi treatment, when most physiological changes with this treatment had waned [24]. Examination of the responses to saline loading, when the responses to early life ACEi were more robust [23], would have strengthened the conclusions that could be drawn. Further, investigation into whether a urinary concentrating defect was present in SFK+ACEi animals would be of interest. Therefore, future studies should examine the responses to water loading and/or dehydration to examine whether the urinary concentrating system is altered by early life ACEi in SFK.

## Conclusion

In conclusion, in the present study a brief period of ACEi in the postnatal period enhanced the suppression of plasma renin in response to saline infusion. In comparison with both SFK and sham sheep, the diuretic response to slow isotonic saline loading was attenuated in SFK sheep exposed to early life ACEi. This indicates that a brief period of ACEi in the postnatal period promotes fluid retention rather than loss in response to a physiological challenge in SFK. Therefore, a brief period of ACEi in the postnatal period in children with SFK may compromise fluid homeostasis and may present a potential caution for the clinical use of early life ACEi. However, further enquiry into the mechanisms driving the blunting of the diuretic and its dissociation from the natriuretic response to saline loading are required.

## Clinical perspectives

- A SFK from birth is associated with early onset of hypertension, albuminuria and kidney disease. Impaired kidney sodium and water handling contributes to hypertension and kidney dysfunction in renal mass reduction. The present study tested if a brief period of ACEi early in life in sheep with SFK improved fluid and sodium excretion in response to saline loading at 20 months of age.
- In response to a slow isotonic saline load, sodium excretion was similar between SFK, sham and SFK sheep that received early life ACEi. Total urine excreted was similar between SFK and sham sheep but ∼40% lower in SFK sheep that received brief early ACEi compared with SFK.
- Brief postnatal ACEi increases fluid retention in response to a physiological challenge in SFK later in life. ACEi early in postnatal life in SFK may compromise fluid homeostasis and may present a potential caution for the clinical use of early life ACEi.

## Supplementary Material

Supplementary Figure S1

## Data Availability

The data underlying this article will be shared on reasonable request to the corresponding author.

## Open Access

Open access for this article was enabled by the participation of Monash University in an all-inclusive *Read & Publish* agreement with Portland Press and the Biochemical Society under a transformative agreement with CAUL.

## Abbreviations

ACEi: angiotensin-converting enzyme inhibition
AngII: angiotensin II
AVP: arginine vasopressin
AQP2: aquaporin 2
BP: blood pressure
ERPF: effective renal plasma flow
GFR: glomerular filtration rate
MAP: mean arterial pressure
MD: macula densa
PAH: p-aminohippuric acid
PRA: plasma renin activity
RAS: renin angiotensin system
RBF: renal blood flow
RVR: renal vascular resistance
SFK: solitary functioning kidney
V2R: vasopressin V2 receptor
