# Supplementary material for: Brief early life angiotensin-converting enzyme inhibition attenuates the diuretic response to saline loading in sheep with solitary functioning kidney
Source: Clin Sci (Lond). 2023 Aug 22;137(16):1285–96. doi: 10.1042/CS20230663 (PMC10447225; doi:10.1042/CS20230663)
Supplement: Supplementary Figure S1 [file CS-2023-0663_supp.pdf]

Supplement Figure 1. Cardiovascular and kidney hemodynamics response to vehicle infusion

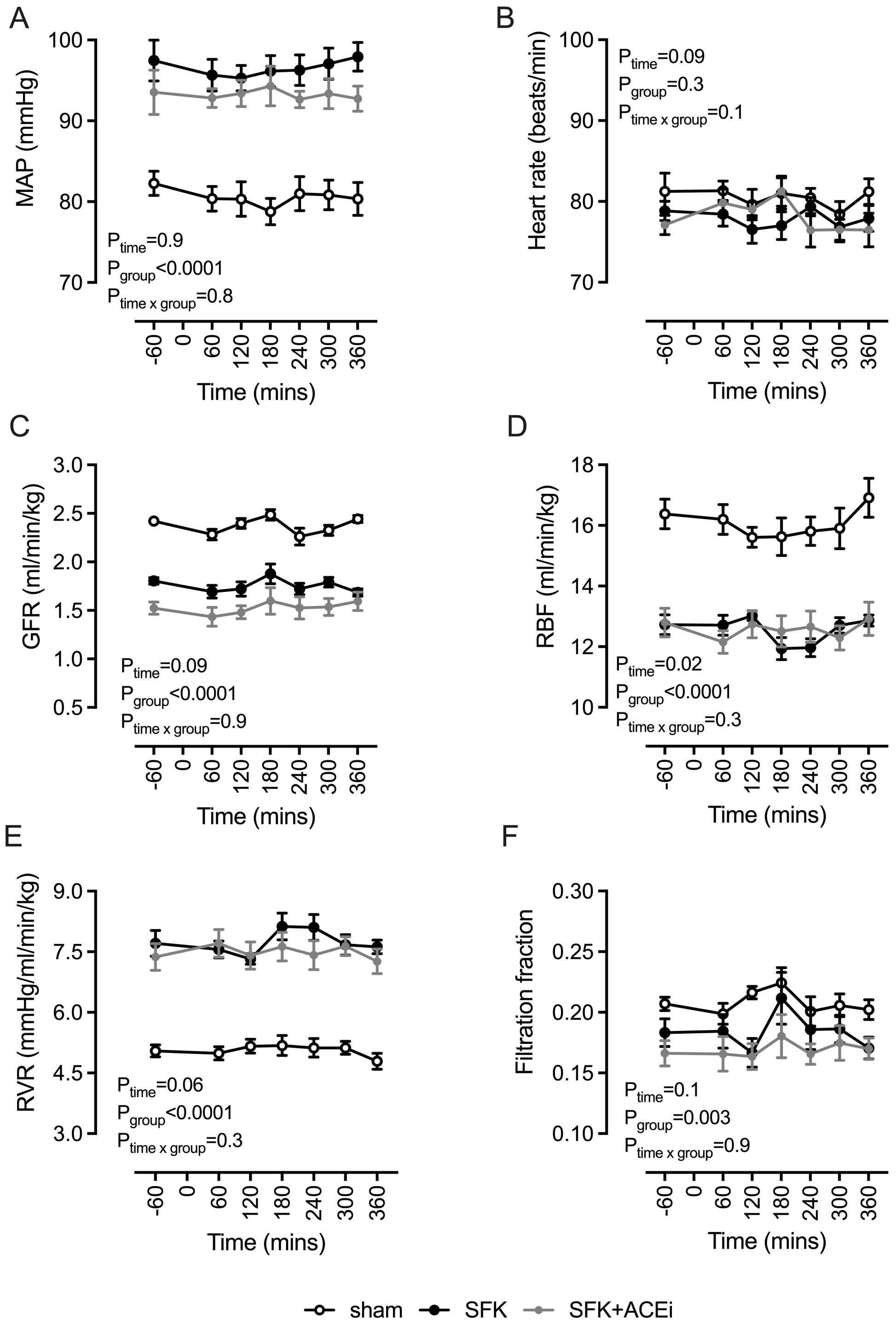

Supplemental Figure 1: Cardiovascular (A,B) and kidney hemodynamics (C-F) during 7 hours of vehicle infusion (time-control). Data presented in male lambs that underwent fetal sham surgery (n=8), fetal uninephrectomy (SFK; n=9) or fetal uninephrectomy and angiotensin converting enzyme inhibition (ACEi) via enalapril between 4-8 weeks of age (SFK+ACEi; n=8). Data were analysed via a two-way repeated measures analysis of variance. MAP; mean arterial pressure, GFR; glomerular filtration rate, RBF; renal blood flow, RVR; renal vascular resistance.
